# How does reasoning influence intentionality attribution in the case of side effects?

**DOI:** 10.1007/s10339-025-01300-w

**Published:** 2025-08-21

**Authors:** Nicola Matteucci Armandi Avogli Trotti, Micaela Maria Zucchelli, Andrea Pavan, Laura Piccardi, Raffaella Nori

**Affiliations:** 1 Department of Psychology, University of Bologna, V.le Berti Pichat, 5, Bologna, Bo 40127 Italy; 2 Department of Psychology, “Sapienza” University of Rome, Rome, RM Italy; 3 IRCCS San Raffaele Cassino, Cassino (FR), Italy

## Abstract

To evaluate others’ actions objectively, one must integrate the actor’s mental states with the potential consequences of his actions. However, consequences can distort the perception of intentionality. The Knobe effect, or “side-effect effect,” demonstrates that individuals attribute greater intentionality to negative than positive foreseen yet unintended side effects. This study explores how reasoning styles and abilities influence these judgments. A sample of 172 college students completed validated reasoning style questionnaires, including the Rational-Experiential Inventory (REI) and the Actively Open-Minded Thinking scale (AOT), a syllogistic reasoning task, and scenario-based tasks in a randomized, between-subjects design (negative vs. positive side effect). Our findings reveal that a more deliberative reasoning style and longer response times both reduce bias in attributing intentionality to negative side effects, highlighting two distinct pathways through which response times mediate the influence of reasoning style on reducing biased judgments. We explore how reasoning affects our attributions of intentionality leading to a more balanced consideration of an actor’s mental state and the consequences in moral judgment.

**Supplementary Information:**

The online version contains supplementary material available at 10.1007/s10339-025-01300-w.

## Introduction

Living in complex societies, human beings have evolved various general cognitive processes to navigate the challenges of intricate social interactions. Humans use these processes when interacting with each other and observing situations from an outsider’s perspective (Buckholtz et al. 2012). Most legal systems rely on judges’ impartial judgment and reasoned decisions (Darley 2009; Robinson et al. 2007). These decisions depend on two important factors: the mental state of the person while acting (*mens rea*) and the consequences of the action (*actus reus*)(Cushman 2008), which influence verdicts and sentences (Shen et al. 2011). Sometimes, *mens rea* and *actus reus* do not align, creating a cognitive challenge. This is especially true in cases of accidental harm, recklessness, or negligence, where evaluators must judge individuals who caused negative outcomes without intent to harm (Margoni and Surian 2022; Patil and Trémolière 2021; Young and Saxe 2009).

The Knobe effect in literature describes how intent (*mens rea*) and action (*actus reus*) influence the perception of intentionality based on the type of outcome (Knobe 2003). The Knobe effect shows that people consider foreseeable but unintended negative side effects of an action to be more intentional than positive ones. This highlights how consequences influence judgments of intentionality, shaping whether an action is seen as more intentional or accidental. The effect is robust across cultures, age groups, and scenarios (Burra and Knobe 2006; Feltz 2007; Leslie et al. 2006). Many studies have tried to understand and explain the imbalance in assigning intentionality between negative and positive side effects, exploring many aspects, such as moral valence, emotional reactions to negative results, and thinking about the actor’s thoughts (Feltz 2007; Knobe 2022; Zucchelli et al. 2018, 2019). Moral valence is one of the most widely accepted theories (Knobe 2003). This theory suggests that moral judgments affect how individuals perceive intentionality. When a side effect is perceived as morally negative, people are more likely to think it was intentional because they believe the person deserves to be blamed for its occurrence. 

Previous research suggests that evaluating accidental harm takes longer than assessing intentional harm, as individuals need extra time to evaluate and integrate conflicting information about mental state (*mens rea*) and consequences (*actus reus*) into their judgments (Buckholtz et al. 2008; Buon et al. 2016; Decety and Cacioppo 2012; Young and Saxe 2009). Dual-Process theories (DPT) offer a framework for understanding these judgments, distinguishing between an intuitive system (System 1) that is fast and emotion-driven, and a deliberative system (System 2) that is slower and based on analytical reasoning (Evans 2008; Kahneman 2011; Sloman 1996). The two-process model of intent-based morality posits that judgments of accidental harm can be seen as the outcome of a cognitive conflict between an outcome-based process (System 1), influenced by emotional reactions to harm severity, and an intent-based process (System 2) which seeks to exculpate the agent by integrating information about their benign mental state (Cushman 2008, 2013; Cushman et al. 2006). Research shows that slower, controlled reasoning can resolve the cognitive conflict in evaluating accidental harm, leading to more lenient moral judgments. Disrupting this process with time constraints or cognitive load results in harsher judgments (Buon et al. 2013; Martin et al. 2021; Schwartz et al. 2024), while individuals with higher rational reasoning style evaluate accidental harm more leniently (Patil and Trémolière 2021; Schwartz et al. 2022). The authors hypothesized that these results arise because better reasoners are more able to prioritize *mens rea* over the more intuitive and emotional *actus reus* information when making judgments. Similarly, also reasoning abilities have been found to be a good predictor of individuals’ capacity to override intuitive and spontaneous responses in accidental harm evaluation (Patil and Trémolière 2021) or moral dilemmas (Patil et al. 2021) following the same pattern described for the rational reasoning style. Ngo et al. (2015) explored the neural basis of the Knobe effect, finding that the amygdala’s higher activity correlates with stronger attributions of intentionality in negative situations, driven by subjective emotional reactions. They suggested that the dlPFC is involved because it plays a role in the cognitive theory of mind, but found no mediating effect on subjective emotional responses. Their study found that participants reacted more quickly to negative scenarios, suggesting that stronger emotional responses drive the Knobe effect. In support of this, Pinillos et al. (2011) found that individuals with higher cognitive reflection abilities exhibited a weaker Knobe effect, suggesting how deliberative reasoning can mitigate its influence.

Building on the existing findings from the literature on moral evaluation of accidental harm, we aim to investigate the potential role of reasoning in diminishing the asymmetry in intentionality attribution seen in the Knobe effect. To do this, we will evaluate participants’ reasoning styles and abilities, as these factors may help alleviate the biases associated with *mens rea* and *actus reus* integration.

Rational reasoning style has been described with different facets and underlying constructs (Baron et al. 2015; Decety and Cacioppo 2012; Thompson & Evans 2012). In the moral field, the Rational-Experiential Inventory (REI; Pacini and Epstein 1999) and the Actively Open Minded Thinking scale (AOT; Stanovich and Toplak 2023; Stanovich and West 1997) are the two most commonly used measures (Trémolière et al. 2017). The REI is based on Epstein’s Cognitive-Experiential Self-Theory (CEST) (Epstein et al. 1996; Pacini and Epstein 1999), grounded in dual-process theories, the REI measures individual preferences for these thinking styles through two subscales that distinguish between two cognitive systems: a fast, intuitive experiential system (measured by REI-E subscale) and a slower, deliberate and rational one (measured by REI-R subscale). The AOT scale (Baron 1993, 2005; Stanovich and Toplak 2023), measures openness to alternative viewpoints, sensitivity to contradictory evidence, and resistance to cognitive closure. It reflects a reflective, analytical, and unbiased reasoning disposition. The construct aims for intellectual flexibility, such as considering opposing evidence, or different points of view, while discouraging dogmatism. While REI-R and AOT both center on rational thought, they differ in scope and emphasis. REI-R focuses on a cognitive disposition toward logic, problem-solving, and detailed analytical examination, whereas AOT pursues a specific style of cognitive regulation aimed at not systematically reinforcing previously acquired beliefs (Baron 2020; Stanovich and Toplak 2023; Trémolière et al. 2017).

Building on previous theories about deliberative processing in moral cognition, we propose that higher-order thinking reduces biases from emotional responses and allows for better evaluations focused on the actor’s mental state. We hypothesize that individuals with stronger deliberative reasoning skills, measured by the REI, AOT, and syllogistic reasoning tasks, will be more effective at integrating *mens rea* and *actus reus* when evaluating scenarios related to the Knobe effect. These individuals should rely less on emotional reactions and more on reflective, analytical thinking, resulting in a decreased tendency to assume intent in negative side effects. This proposed mechanism aligns with previous studies showing that deliberate reasoning can counter biased responses in moral judgments based on intent (Patil and Trémolière 2021; Schwartz et al. 2022). Moreover, we predict that response times will further reveal this dynamic: individuals who spend more time reasoning through the Knobe scenarios and engaging with the information in depth will attribute lower levels of intentionality to negative side effects compared to those who respond quickly. This aligns with the idea that slower, deliberative processes reduce reliance on automatic, emotionally driven judgments.

In summary, our key prediction is that individuals who engage in more deliberative thinking are likely to display reduced bias in scenarios related to the Knobe effect, particularly in terms of attributing intentionality to negative side effects. This leads to two main conclusions: (i) More deliberative reasoning reduces reliance on emotions and intuition; (ii) Emphasizing analytical thinking promotes a balanced view of both intent and outcomes.

## Materials and methods

### Participants

Our sample comprised 172 participants (GPower 3.1. Faul et al. 2007, 2009; f2 = 0.25, α = 0.05, Power = 0.90), recruited at University and from citizen associations through notices on social networks and bulletin boards (age M = 21.75, SD = 2.14; 31% males; education M = 14.89, SD = 2.57). Exclusion criteria included a history of neurological or psychiatric disorders, as well as the use and abuse of substances. No participants were excluded. All participants signed a written consent form before the study commenced. The local Ethics Committee approved the study (Prot. n. 130861, University of Bologna, Italy).

### Task and materials

All materials and tasks related to the experiment are detailed in the supplementary section of this paper. Additionally, the data presented in this study can be accessed through the OSF repository at the following link:

https://osf.io/cvj5y/?view_only=b41ffd2cf3a542bb8fc6091c7fafe185.

#### Knobe effect scenario (Ngo et al. 2015; Zucchelli et al. 2018, 2019)

We selected eight negative and eight paired positive scenarios from prior research on the Knobe effect using the same set of stimuli adopted by Zucchelli et al. (2018, 2019), and drawing from the eighty scenarios identified by Ngo et al. (2015). This selection was made because these stimuli have proven effective and sensitive in eliciting differences in intentionality attribution in previous studies employing similar designs. Moreover, it promotes methodological consistency and comparability with earlier research. Each scenario depicts a character’s action that leads to an unintended but anticipated consequence (see supplementary material for the complete set). Following each scenario, participants rated the intentionality of the side effect on a Likert scale from 0 (not intentional at all) to 7 (completely intentional).

#### Rational experiential inventory (REI; Pacini and epstein 1999)

The REI was used to assess participants’ reliance on two main modes of thinking: fast, intuitive, automatic thinking and slower, logical thinking. This experiment analyzed 20 items from the REI-R subscale related to rational thinking, such as “*I have no problem thinking things through carefully*”. Participants rated how well each item described them on a 5-point scale (1 = definitely not true of myself, 5 = definitely true of myself). Higher scores indicated a greater degree of rationality. Several studies have provided evidence of good test reliability: *Cronbach’alpha* = 0.86 to 0.90 (Björklund and Bäckström 2008; Pacini and Epstein 1999; Sladek et al. 2010; Witteman et al. 2009).

#### The actively Open-Minded thinking scale (AOT; Baron et al. 2015; Baron 2019)

This scale evaluates participants’ tendencies toward open-minded thinking. As described in the introduction, this includes their willingness to fairly consider diverse conclusions, even those contradicting their initial preferences; their careful consideration of others’ viewpoints when forming their own opinions; and their general propensity to actively and openly contemplate available information. Items on the AOT scale capture attitudes such as “*People should always take into consideration evidence that goes against their beliefs*” and “*Allowing oneself to be convinced by a solid opposing argument is a sign of good character*”. Participants respond on a 5-point scale, from 1 = “*Completely disagree*” to 5 = “*Completely agree*”, with respect to each of the eight items. Higher scores indicate a greater tendency toward an open-minded thinking style. The scale demonstrated good reliability, with Cronbach’alpha = 0.65 to 0.92 (Baron 2019; Janssen et al. 2020; Stanovich and Toplak 2023).

#### Belief bias (BB; Evans et al. 1983)

This task measures reasoning abilities and shows the conflict between intuitive processes, which favor belief, and deliberative processes, which prioritize logical structure. In a typical syllogistic task, participants evaluate arguments where the logical validity of the conclusion does not align with its intuitive plausibility, thus assessing logical reasoning and one’s capacity to resist cognitive biases. This makes it a valuable tool for understanding the interaction between rational and intuitive cognitive processes. In a conflict syllogism, conclusions are logically invalid but believable, as in the following example:

*All quadrupeds are mammals.*

*All quadrupeds are animals.*

*Therefore*,* all mammals are animals.*

The ability to solve conflict syllogisms is closely related to higher reasoning abilities and engagement in reflective thinking (Baron et al. 2015; Evans et al. 1983; Klauer et al. 2000; Thompson & Evans 2012). We used eight conflict syllogisms taken from previous studies on belief bias in syllogistic reasoning (Morley et al. 2004; Thompson & Evans 2012). Participants judged whether the conclusion logically followed using a binary YES/NO response. Participants received 1 point for each correctly solved syllogism, with higher scores indicating lower belief bias (i.e., greater reliance on analytical reasoning).

### Procedure

The experiment was administered online through the software Qualtrics (First release: 2005, Provo, Utah, USA, available at: https://www.qualtrics.com*).* After providing demographic information, participants completed the experiment in a randomized order. Specifically, half of the participants first answered the Knobe Effect questions, and then completed the reasoning questionnaires (REI, AOT, and syllogisms). The other half did the reasoning questionnaires first, followed by the Knobe scenarios. The order of items in both the scenarios and reasoning questionnaires was randomized to prevent order effects. Participants were randomly assigned to one of two main conditions: negative side effects or positive side effects (*between-subjects design*; Zucchelli et al. 2019). Each scenario appeared across two screens: the first described the context and actions, while the second asked participants to rate the side effect’s intentionality on a 7-point Likert scale (0 = *not intentional at all*, 7 = *completely intentional*). This design accurately measured response times for the intentionality question before confirming answers. The procedure was the same for all of the 8 scenarios for each participant. The experiment lasted approximately 30 min.

### Statistical analysis

For each participant, we calculated the mean of intentionality attribution scores and response times. Initially, we tested for differences in intentionality attribution and response times between negative and positive scenarios using independent-samples *t*-tests. This was done to assess the presence of the Knobe effect in the selected scenarios and to compare reasoning times between the two types of scenarios. Next, we performed two mediation models - one for negative and one for positive scenarios - with reasoning measures as predictors and response times as mediators. We aimed to determine how reasoning influences intentionality attribution differently in negative and positive scenarios. Our analysis aimed to determine whether different facets of reflective thinking affect the integration of *mens rea* and *actus reus* over time. The analyses were conducted using JASP (version 0.17.2.1).

## Results

There were no significant differences in demographic variables or reasoning skills and abilities between the two groups (see supplementary materials, *ps* > 0.05). We tested the normality of the residuals for the intentionality attribution scores using the Shapiro-Wilk test. The results were significant (Shapiro-Wilk = 0.94, *p* < .001), indicating some deviation from normality. The skewness and kurtosis were − 0.67 (*SE* = 0.185) and − 0.37 (*SE* = 0.37), respectively. Given the deviation from normality suggested by the Shapiro-Wilk test, we used the Mann-Whitney U test to examine the differences between the two conditions. We found that negative side effects (*M* = 5.23, *SD* = 0.98) were perceived as significantly more intentional than positive ones (*M* = 2.82, *SD* = 1.56) (Mann Whitney *U* = 628, *p* < .001, *Rank-Biserial Correlation* = 0.83). Besides, response times were significantly faster for negative side effects (*M* = 3.57 s, *SD* = 3.44 s) compared to positive ones (*M* = 4.32 s, *SD* = 2.23 s) (Mann-Whitney *U* = 2380, *p* < .001, *Rank-Biserial Correlation* = 0.36).

### Negative scenarios mediation model

All the other measures significantly correlated with each other, except for syllogisms (see Table 1), which were not found to predict intentionality attribution and were therefore excluded from the model.

**Table 1:** Pearson’s correlation coefficients

	Intentionality scores	Reaction times	AOT	REI	Syllogisms
Intentionality scores	-				
Reaction times	− 0.411***	-			
AOT	− 0.319**	0.241*	-		
REI	− 0.335**	0.330*	0.256*	-	
Syllogism	− 0.200	0.149	0.089	0.061	-

*** *p* < .001, ** *p* < .01, * *p* < .05

We consequently performed a mediation model with REI and AOT scores as predictors, the mean of response times as mediating factor, and the mean of intentionality attribution as the dependent variable. We used bootstrapping (1,000 samples with 95% bias-corrected confidence intervals) as a non-parametric resampling procedure to estimate the significance of the indirect effect. A significance level of 0.05 was used to determine the statistical significance of both direct and indirect paths.

**Fig. 1:**
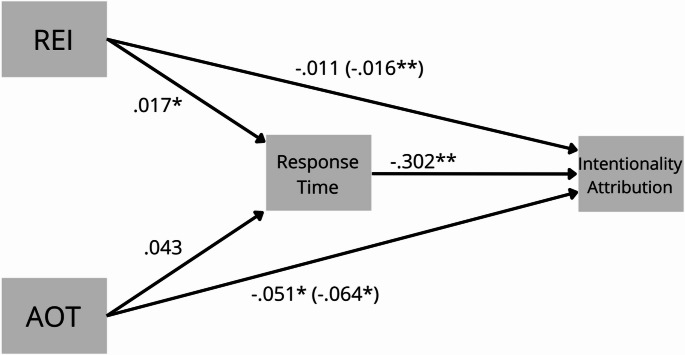
Mediation model showing the full mediation of response times between REI scores and intentionality attribution. Values are bootstrapped path coefficients, with total effects shown in brackets. * *p* < .05, **p < .01

The total effect of REI and AOT were both significant (*β* = –0.016, *p* < .01, 95% CI [–0.031, –0.001]) and (*β* = − 0.064, *p* < .05, 95% CI [–0.115, 8.2 × 10^− 4^], respectively. The mediation analysis showed that response times significantly and fully mediated the effect of REI scores on intentionality judgments for negative side effects (*β* = –0.005, *p* < .05, 95% CI [–0.015, − 5.439 × 10^− 4^]). However, this was not the case for the AOT scores, where no significant mediation was observed (*β* = –0.013; *p* = .156, 95% CI [–0.049, 0.003]). The mediation for the REI scores was complete, as the direct effect of REI on intentionality judgments become not significant when response times were included in the model (*β* = –0.011; *p* > .05, 95% CI [–0.024, 0.002]). Additionally, we observed a negative relationship in the direct path from intentionality attribution to response times (*β* = –0.302; *p* < .01, 95% CI [–0.632, –0.072]) (Fig. 1).

### Positive scenarios mediation model

No significant relationships were found between reasoning abilities, positive side effects, intentionality judgments, and response times.

## Discussion

The present study aimed to examine how reasoning style and abilities modulate the influence of *actus reus* (the consequences of an action) on *mens rea* (mental states/intentions) when attributing intentionality to foreseeable but unwilled positive or negative side effects (i.e. the Knobe effect). We hypothesized that people with stronger reasoning skills would attribute less intentionality to negative side effects, suggesting that those who engage in deeper reflection are more likely to focus on the absence of intention in the action itself, rather than on the negative outcomes.

Our results confirmed the presence of the Knobe effect, with higher intentionality attribution toward negative side effects (Knobe 2003). Consistent with previous findings, we also observed shorter response times when participants evaluated negative compared to positive side effects (Ngo et al. 2015). This faster response to negative side effects may reflect an emotional response triggered by the negative consequences (Nadelhoffer 2004; Ngo et al. 2015; Zucchelli et al. 2019). Relying on emotions, participants likely responded more intuitively, predominantly engaging System 1 processes. This would result in a simplified mental model that relies more on consequences than on intentions, as postulated by the two-process model of moral judgment (Cushman 2008). Similarly, when participants are forced to rely on System 1 - such as under time pressure or cognitive load (Buon et al. 2013; Martin et al. 2021; Schwartz et al. 2024) - they tend to place more emphasis on consequences rather than intentions when evaluating accidental harm scenarios.

Regarding our main hypotheses, the results confirmed that both an open-minded reasoning style (AOT) and a rational reasoning style (REI-R) were associated with lower intentionality attribution to negative side effects. Besides, longer response times, indicating more extended reasoning to give their response, showed the same pattern. These three measures also correlate with each other. A higher propensity for rational thinking has been linked to less harsh judgements of accidental harm (Patil and Trémolière 2021; Schwartz et al. 2022), consistent with our findings that this propensity facilitates a more accurate consideration of *mens rea.* Thus, our results, along with previous evidence, suggest that a rational reasoning style can influence how people integrate intent-based and outcome-based judgments in moral decision-making (Cushman et al. 2006; Schwartz et al. 2022).

Interestingly, our study demonstrates that reasoning may directly influence the perception of intentions in case of negligence (i.e., foreseen but unintended consequences), whereas previous research focused primarily on moral judgments such as punishment, moral acceptability, or culpability, and has only considered cases of pure accidental harm (Buon et al. 2013; Martin et al. 2021; Patil and Trémolière 2021; Schwartz et al. 2022). Our results suggest that reasoning may influence how we evaluate events by shaping our perception of intentions even before affecting later judgments.

Although our study did not directly assess perceptions of negligence, the scenarios employed, in which agents foresee negative side effects yet choose to act regardless, represent clear examples of negligent behavior, where foreseeable consequences are knowingly disregarded. Indeed, definitions of negligence (e.g. Nũnez et al. 2014; Flick et al. 2024), according to the Malle and Knobe’s (1997) model of intentionality, consistently incorporate features of ‘knowledge’ and ‘awareness’, meaning that the actor knew or was aware of the potential consequences of their actions, but do not include the ‘desire’ to bring about negative outcomes, just like in the Knobe’s negative scenarios (Guglielmo and Malle 2010).

Our results are the first to show that reasoning style can directly influence perceived intentionality in such contexts, rather than merely affecting subsequent moral evaluations. Previous literature has, in fact, primarily focused on the role of reasoning in generating more lenient judgments in cases of purely accidental harm, without explicitly manipulating or measuring negligence (Patil and Trémolière 2021; Schwartz et al. 2022).

Nonetheless, our findings may offer even greater interpretive value in light of recent studies highlighting the spontaneous inferences of recklessness and negligence that often arise when evaluating classically accidental scenarios.

Research shows that people often attribute negligence when only intentions and outcomes are known and no explicit information is provided regarding foreseeability or care (Nobes and Martin 2022). Moreover, even describing someone as acting carefully yet still causing harm can lead individuals to infer that the agent should have foreseen the harmful consequences of their actions, thereby inferring negligence in the absence of specific information (Margoni and Surian 2022). These findings are consistent with developmental and lifespan research showing that both young children and older adults tend to over-attribute negligence in response to accidental negative outcomes, particularly when information about care or foreseeability is missing or underspecified (Margoni et al. 2019; Nobes et al. 2009, 2017). It has been suggested that properly processing and integrating information about *mens rea* requires substantial cognitive effort, which may be more challenging for these population groups due to underdeveloped or declining executive function skills (Margoni et al. 2018, 2019; Margoni and Surian 2016).

In light of our findings on reasoning propensity and intentionality attribution, we speculate that better reasoners may judge accidental harm more leniently not only because they are less influenced by outcomes in their moral evaluations, but also because, even when considering different possible levels of care and foreseeability, they ultimately perceive the action as less intentional, and thus intrinsically less blameworthy or less deserving of punishment.

Our study, which directly measured intentionality attribution rather than moral or punishment judgments in response to negative and foreseeable outcomes, combined with individual differences in reasoning, offers a novel perspective on the role of reasoning in intent-based moral judgment. Given that previous research has shown how negligence is often inferred in evaluations of accidental harm, the mechanism identified in our findings may also apply to such cases.

Specifically, better reasoners may be more likely to perceive actions involving possible negligence and a negative outcome as less intentional from the outset, rather than simply focusing less on the outcome during the construction of punishment and moral judgments.

Our results thus contribute, through the combination of reasoning propensity measures and intentionality attribution, to adding a new piece to the puzzle of intent-based moral judgment and reasoning. They suggest that deliberative thinking can act directly on a core antecedent of moral evaluation, namely perceived intentionality (Cushman 2015), reflecting the idea, widely discussed in the Knobe effect literature, that judgments of intentionality and moral evaluation may not follow a fixed or strictly sequential order, but can interact and shape each other in dynamic ways (Feltz 2007; Leslie et al. 2006; Pettit and Knobe 2009).

Our mediation analysis, which considered REI-R and AOT as predictors and response times as a mediator, revealed important distinctions between these two aspects of rational thinking. For REI-R, longer response times fully mediated the attribution of intentionality, indicating that reflective, deliberate reasoning plays a key role in diminishing the Knobe effect. In contrast, for AOT, response times did not significantly mediate effect, suggesting that open-minded thinking operates independently of prolonged deliberation when modulating biased responses in the Knobe effect. These findings highlight distinct cognitive mechanisms through which individuals with high REI-R and AOT scores reduce the Knobe effect. Although correlated, REI-R and AOT represent different facets of rational reasoning, operating through different mechanisms. Prior studies confirm this distinction, showing that despite their correlation, they influence cognition and behavior in distinct ways (MacLaren et al. 2012; Ozono & Sakakibara 2024; Svedholm-Häkkinen and Lindeman 2017; Szaszi et al. 2017).

Individuals with high REI-R scores rely on reflective, analytical reasoning, aligning with System 2 processes (Kahneman 2011; Stanovich and West 2000). This resource-intensive approach involves deconstructing information, weighing evidence, and suppressing initial intuitive responses - especially in emotionally charged contexts like the Knobe effect. This deliberate method of conflict resolution could clarify why participants with high REI-R took longer to diminish their biases. Their reasoning style, which prioritizes constructing rational, well-founded judgments, led them to focus more on the agent’s lack of intent, rather than being mainly influenced by the negative consequences.

In contrast, individuals with high AOT scores adopt a flexible, integrative reasoning style, characterized by openness to different perspectives and a readiness to revise conclusions based on new information (Stanovich and Toplak 2023; Svedholm-Häkkinen and Lindeman 2017). They are more adept at processing conflicting information early, distinguishing foreseen but unintended consequences from fully intended actions. This ability likely explains why their judgments were less dependent on response time. The key difference between these reasoning styles lies in their approach; high REI-R involves effortful conflict resolution, whereas high AOT reflects a more dynamic integration. While high REI-R individuals process information in a way that resembles the traditional dual-process framework, where System 2 overrides System 1 (Evans 2003; Evans & Stanovich 2013), high AOT individuals reflect a more integrative model, where intuitive and logical reasoning coexist dynamically, enabling them to form balanced judgments more quickly. This aligns with recent dual-process theories, which suggest that logical responses can occur intuitively without the need for explicit intervention of System 2 intervention (Bago and De Neys 2019a; Bago and Neys 2019b; De Neys and Pennycook 2019). Both reasoning styles, however, contribute to reducing the Knobe effect bias, albeit through distinct cognitive pathways.

Our findings offer insights into the coexistence of two hypothesized mechanisms within the brain’s central executive network that regulate *mens rea* and *actus reus* information (Krueger and Hoffman 2016; Yang et al. 2024). Specifically, the Integration and Selection Hypothesis (Buckholtz et al. 2008, 2012), suggests that the dlPFC integrates and selects relevant information during judgment, aligning with the strategy used by high AOT individuals. Conversely, the Cognitive Control Theory (Knoch et al. 2006; Spitzer et al. 2007) proposes that the dlPFC exerts top-down regulation to suppress emotional responses, consistent with the deliberate, step-by-step reasoning observed in high REI-R individuals. We did not find any significant relationship between syllogistic reasoning abilities and intentionality attribution for negative side effects. This suggests that REI-R and AOT may better capture the reasoning required to resolve cognitive conflicts between intentions and consequences in moral judgments. Unlike syllogistic reasoning, which focuses on logical validity without emotional content, intentionality attribution in the Knobe effect involves integrating emotionally charged, context-specific information about *mens rea* and *actus reus*, while managing intuitive reactions. As a result, syllogistic reasoning may be less effective in predicting the ability to inhibit intuitive and emotional responses in the Knobe effect and properly integrate morally relevant information. The absence of a moral or social dimension in syllogistic reasoning may limit its applicability to real-world scenarios that require complex emotional and theory of mind-based reasoning.

Interestingly, none of our reasoning measures were significantly related to judgments of positive side effects. This may be because positive consequences without specific intent involve a simpler evaluative process, requiring less effort to inhibit intuitions or resolve conflicting information. Consequently, higher reasoning abilities or stronger regulation of intuitive responses do not seem to influence intentionality attribution in these cases. The contrast between negative and positive side effects underscores the distinct cognitive and emotional processes involved. As previously suggested (Ngo et al. 2015; Pinillos et al. 2011), negative side effects elicit stronger intuitive, emotionally driven responses, mediated by System 1. Individuals with a greater propensity for rational thinking are better equipped to regulate these intuitions, which explains their ability to modulate intentionality attribution in negative scenarios.

Our findings are the first to demonstrate that two distinct forms of rational thinking can lead to similar outcomes in moral judgment through different cognitive pathways. This distinction has important implications for experimental paradigms that rely on manipulations such as time pressure or cognitive load to influence reasoning processes (Buon et al. 2013; Martin et al. 2021; Zucchelli et al. 2025). Specifically, it suggests that individuals with different reasoning profiles may be differentially susceptible to such manipulations. Furthermore, our results help deepen our understanding of dual-process theories on intent-based moral judgment, showing how participants with a preference for slow and open reasoning are not only less influenced by negative outcomes in moral judgment (Patil and Trémolière 2021; Schwartz et al. 2022), but also less influenced by them when it comes to perceiving the intentions behind the action. As stated above, this opens new ways of interpreting the role of reasoning in intent-based moral judgment, and raises new interesting research questions regarding the impact of reasoning on the perception of intention, consequences, and consequent moral judgments.

### Limitations and future research

While our findings provide valuable insights into the cognitive mechanisms underlying intentionality attribution, some limitations should be noted. First, our reliance on REI-R and AOT may not fully capture the interplay between reasoning, emotional regulation, and intentionality judgments. Specifically, these measures assess general reasoning dispositions but do not directly evaluate cognitive processes such as inhibitory control, which could play a crucial role in intentionality decision-making. Future studies could incorporate additional specific measures, such as classic and emotional Stroop tasks (Greco 1993; Stroop 1935) for measuring inhibition abilities or controls for subjective emotional responses. Second, we did not manipulate reasoning directly (e.g., through cognitive load or time pressure), which limits our ability to establish a causal link between reasoning and intentionality attribution. Incorporating such manipulations could refine our understanding of the temporal dynamics of reasoning in this process. Methods like the two-response paradigms (Bago and De Neys 2019a; De Neys and Pennycook 2019; Trémolière et al. 2017), could help address this gap. Third, the null results for belief bias syllogisms suggest the need for measures that better reflect the emotional and cognitive demands of the task. In fact, we manipulated only the type of consequences in our scenarios. Considering emotional factors and different levels of conflicting information in problem-solving can help identify when reasoning skills are most influential. Future research should explore how reasoning influences judgments in cases of accidental harm, by controlling for perceived intentionality and foreseeability. This would help differentiate between truly accidental harm and harm caused by recklessness or negligence. Expanding these methodological approaches will provide a more comprehensive understanding of the interaction between reasoning and intuitive processes in intent-based moral judgment.

## Conclusions

In summary, the present findings highlight the intricate nature of integrating *mens rea* and *actus reus* information in human cognition. In instances where these components interact, as evidenced by the Knobe effect, it becomes imperative to engage higher-order cognitive processes to regulate strong, automatic intuitive and emotionally driven reactions, or to selectively prioritize and integrate all pertinent information from the outset. The observed differences in reasoning strategies demonstrate how distinct cognitive pathways - such as deliberate, reflective suppression and flexible, integrative reasoning - can lead to similar outcomes, namely, reducing the attribution of intentionality to negative side effects.

## Supplementary Information

Below is the link to the electronic supplementary material.

Supplementary Material 1
