# Supplementary material for: How does reasoning influence intentionality attribution in the case of side effects?
Source: Cogn Process. 2025 Aug 21;27(1):109–19. doi: 10.1007/s10339-025-01300-w (PMC12860760; doi:10.1007/s10339-025-01300-w)
Supplement: Supplementary file 1 — Supplementary Material 1 [file 10339_2025_1300_MOESM1_ESM.docx]

**Supplementary material**

1. **Knobe effect scenarios**
   1. **Negative:**

1. The company's director has initiated an industrial plan to increase profits. He knew the industrial plan would harm the environment, but he didn't care.

Did the director intentionally harm the environment?

2. The mayor has diverted the river's water to the city of Trombacco to gain more votes. He knew this would deprive the city of Barga of water, but he didn't care.

Did the mayor intentionally deprive Barga of water?

3. The pharmaceutical company has released a new drug to increase profits. They knew the drug would increase the rate of vascular disorders, but they didn't care.

Did the pharmaceutical company intentionally increase the rate of vascular disorders?

4. The mayor has cut expenses at the animal shelter to increase the salaries of municipal executives. He knew this would result in the closure of the animal shelter, but he didn't care.

Did the mayor intentionally cause the closure of the animal shelter?

5. The school principal has adopted a new meal plan to cut cafeteria costs. He knew the new meal plan would harm the children's health, but he didn't care.

Did the school principal intentionally harm the children's health?

6. The government has reorganized the Ministry of Education's finances to balance the budget. They knew the new financial organization would harm education, but they didn't care.

Did the government intentionally harm education?

7. The farmer has decided to cultivate a new type of GMO tomato to increase sales. He knew the new GMO tomato would make their customers sick, but he didn't care.

Did the farmer intentionally cause illness in their customers?

8. The doctor has prescribed a medication to appease the pharmaceutical company. He knew the medication would cause inheritable genetic alterations in those who take it, but he didn't care.

Did the doctor intentionally cause inheritable genetic alterations in those who take the medication?

- 1. **Positive:**

1. The company's director has initiated an industrial plan to increase profits. He knew the industrial plan would help the environment, but he didn't care.

Did the director intentionally help the environment?

2. The mayor has diverted the river's water to the city of Trombacco to gain more votes. He knew this would increase food production, but he didn't care.

Did the mayor intentionally increase food production?

3. The pharmaceutical company has released a new drug to increase profits. They knew the drug would decrease the rate of vascular disorders, but they didn't care.

Did the pharmaceutical company intentionally decrease the rate of vascular disorders?

4. The mayor has cut expenses at the animal shelter to increase the salaries of municipal executives. He knew this would result in significant donations from private individuals to help the animals, but he didn't care.

Did the mayor intentionally help the animal shelter obtain more donations from private individuals?

5. The school principal has adopted a new meal plan to cut cafeteria costs. He knew the new meal plan would improve the children's health, but he didn't care.

Did the school principal intentionally improve the children's health?

6. The government has reorganized the Ministry of Education's finances to balance the budget. They knew the new financial organization would improve education, but they didn't care.

Did the government intentionally improve education?

7. The farmer has decided to cultivate a new type of GMO tomato to increase sales. He knew the new GMO tomato would improve the health of their customers, but he didn't care.

Did the farmer intentionally improve the health of their customers?

8. The doctor has prescribed a medication to appease the pharmaceutical company. He knew the medication would cure those who take it, but he didn't care.

Did the doctor intentionally cure those who take the medication?

1. **Reasoning scales**

**2.1. REI-R**

1. I try to avoid situations that require thinking in depth about something
2. I'm not that good at figuring out complicated problems
3. I enjoy intellectual challenges
4. I am not very good at solving problems that require careful logical analysis
5. I don't like to have to do a lot of thinking
6. I enjoy solving problems that require hard thinking
7. Thinking is not my idea of an enjoyable activity
8. I am not a very analytical thinker
9. Reasoning things out carefully is not one of my strong points
10. I prefer complex problems to simple problems
11. Thinking hard and for a long time about something gives me little satisfaction
12. I don't reason well under pressure
13. I am much better at figuring things out logically than most people
14. I have a logical mind
15. I enjoy thinking in abstract terms
16. I have no problem thinking things through carefully
17. Using logic usually works well for me in figuring out problems in my life
18. Knowing the answer without having to understand the reasoning behind it is good enough for me
19. I usually have clear, explainable reasons for my decisions
20. Learning new ways to think would be very appealing to me

**2.2 AOT**

1. Allowing oneself to be convinced by an opposing argument is a sign of good character.
2. People should take into consideration evidence that goes against their beliefs.
3. People should revise their beliefs in response to new information or evidence.
4. Changing your mind is a sign of weakness.
5. Intuition is the best guide in making decisions.
6. It is important to persevere in your beliefs even when evidence is brought to bear against them.
7. One should disregard evidence that conflicts with one’s established beliefs.
8. People should search actively for reasons why their beliefs might be wrong

**2.3 Syllogisms (BB)**

All flowers have petals.

Roses have petals.

Therefore, roses are flowers.

Does the conclusion logically follow from the premises?

All wives are married.

Some women are married.

Therefore, some women are wives.

Does the conclusion logically follow from the premises?

All things that have an engine need oil.

Automobiles need oil.

Therefore, automobiles have an engine.

Does the conclusion logically follow from the premises?

Untrained dogs are vicious.

Some ferocious dogs are police dogs.

Therefore, some trained dogs are not police dogs.

Does the conclusion logically follow from the premises?

All living things need water.

Roses need water.

Therefore, roses are living beings.

Does the conclusion logically follow from the premises?

No addictive thing is cheap.

Some cigarettes are cheap.

Therefore, some addictive things are not cigarettes.

Does the conclusion follow logically from the premises?

1. **Table of correlation for positive side effects and reasoning measures**

*Table 1. Pearson’s correlation coefficients*

|  | Intentionality scores | Reaction times | AOT | REI | Syllogisms |
| --- | --- | --- | --- | --- | --- |
| Intentionality scores | - |  |  |  |  |
| Reaction times | -0.001 | - |  |  |  |
| AOT | -0.017 | -0.040 | - |  |  |
| REI | -0.020 | 0.036 | .258** | - |  |
| BB | 0.050 | 0.087 | .041 | .071 | - |

**** p < .001, ** p< .01, *p< .05*

1. **Testing Differences in Demographics and Measures Between Negative and Positive Groups**

**4.1. Independent sample T-tests for reasoning measures, age and years of education between the two experimental groups**

| **Independent Samples T-Test** | | | | | | | | | | | | | |
| --- | --- | --- | --- | --- | --- | --- | --- | --- | --- | --- | --- | --- | --- |
|  | | *Test* | | *Statistic* | | *df* | | *p* | | *Effect Size* | | *SE Effect Size* | |
| age |  | Student |  | 0.888 |  | 170 |  | 0.376 |  | 0.135 |  | 0.153 |  |
|  |  | Mann-Whitney |  | 4049.000 |  |  |  | 0.277 |  | 0.095 |  | 0.088 |  |
| education |  | Student |  | 0.622 |  | 170 |  | 0.535 |  | 0.095 |  | 0.153 |  |
|  |  | Mann-Whitney |  | 3909.000 |  |  |  | 0.446 |  | 0.057 |  | 0.088 |  |
| AOT |  | Student |  | -0.149 |  | 170 |  | 0.881 |  | -0.023 |  | 0.153 |  |
|  |  | Mann-Whitney |  | 3603.500 |  |  |  | 0.772 |  | -0.026 |  | 0.088 |  |
| REI_R |  | Student |  | 0.377 |  | 170 |  | 0.707 |  | 0.057 |  | 0.153 |  |
|  |  | Mann-Whitney |  | 3810.500 |  |  |  | 0.731 |  | 0.030 |  | 0.088 |  |
| BB |  | Student |  | -0.371 |  | 170 |  | 0.711 |  | -0.057 |  | 0.153 |  |
|  |  | Mann-Whitney |  | 3549.000 |  |  |  | 0.639 |  | -0.040 |  | 0.088 |  |
|  | | | | | | | | | | | | | |
| *Note.*  For the Student t-test, effect size is given by Cohen's d. For the Mann-Whitney test, effect size is given by the rank biserial correlation. | | | | | | | | | | | | | |

- 1. **Contingency table and Chi squatted test for gender distribution in the two experimental groups**

| Contingency Tables (for gender differences) | | | | | | | |  |  |
| --- | --- | --- | --- | --- | --- | --- | --- | --- | --- |
|  | Gender | | | |  | | |  |  |
| Valence | Male | | Female | | Total | | |  |  |
| Positive |  | 30 |  | 56 | |  | 86 | |  |
| Negative |  | 24 |  | 62 | |  | 86 | |  |
| Total |  | 54 |  | 118 | |  | 172 | |  |
|  | | | | | | | |  |  |

| Chi-Squared Tests (for gender differences) | | | | | | | |
| --- | --- | --- | --- | --- | --- | --- | --- |
|  | | Value | | df | | p | |
| Χ² |  | 0.972 |  | 1 |  | 0.324 |  |
| N |  | 172 |  |  |  |  |  |
|  | | | | | | | |
